# Foley catheter vs. oral misoprostol to induce labour among hypertensive women in India: a cost‐consequence analysis alongside a clinical trial

**DOI:** 10.1111/1471-0528.15285

**Published:** 2018-06-22

**Authors:** S Leigh, P Granby, A Haycox, S Mundle, H Bracken, V Khedikar, J Mulik, B Faragher, T Easterling, MA Turner, Z Alfirevic, B Winikoff, AD Weeks

**Affiliations:** ^1^ University of Liverpool Management School Liverpool UK; ^2^ Department of Obstetrics & Gynaecology Government Medical College Nagpur India; ^3^ Gynuity Health Projects New York NY USA; ^4^ Daga Memorial Women's Government Hospital Nagpur India; ^5^ Medical Statistics Unit Department of Clinical Sciences Liverpool School of Tropical Medicine Liverpool UK; ^6^ Department of Obstetrics and Gynecology University of Washington Seattle WA USA; ^7^ Department of Women's and Children's Health University of Liverpool Liverpool Women's Hospital Liverpool UK

**Keywords:** Cost‐consequence, economics, hypertension, labour induction, low‐resource settings, pre‐eclampsia

## Abstract

**Objective:**

To determine the effectiveness and economic impact of two methods for induction of labour in hypertensive women, in low‐resource settings.

**Design:**

Cost‐consequence analysis of a previously reported multicentre, parallel, open‐label randomised trial.

**Setting & population:**

A total of 602 women with a live fetus, aged ≥18 years requiring delivery for pre‐eclampsia or hypertension, in two public hospitals in Nagpur, India.

**Methods:**

We performed a formal economic evaluation alongside the INFORM clinical trial. Women were randomised to receive transcervical Foley catheterisation or oral misoprostol 25 mcg. Healthcare expenditure was calculated using a provider‐side microcosting approach.

**Main outcome measures:**

Rates of vaginal this delivery within 24 hours of induction, healthcare expenditure per completed treatment episode.

**Results:**

Induction with oral misoprostol resulted in a (mean difference) $20.6USD reduction in healthcare expenditure [95% CI (−) $123.59 (−) $72.49], and improved achievement of vaginal delivery within 24 hours of induction, mean difference 10% [95% CI (−2 to 17.9%), *P* = 0.016]. Oxytocin administration time was reduced by 135.3 minutes [95% CI (84.4–186.2 minutes), *P* < 0.01] and caesarean sections by 9.1% [95% CI (1.1–17%), *P* = 0.025] for those receiving oral misoprostol. Following probabilistic sensitivity analysis, oral misoprostol was cost‐saving in 63% of 5,000 bootstrap replications and achieved superior rates of vaginal delivery, delivery within 24 hours of induction and vaginal delivery within 24 hours of induction in 98.7%, 90.7%, and 99.4% of bootstrap simulations. Based on univariate threshold analysis, the unit price of oral misoprostol 25 mcg could feasibly increase 31‐fold from $0.24 to $7.50 per 25 mcg tablet and remain cost‐saving.

**Conclusion:**

Compared to Foley catheterisation for the induction of high‐risk hypertensive women, oral misoprostol improves rates of vaginal delivery within 24 hours of induction and may also reduce costs. Additional research performed in other low‐resource settings is required to determine their relative cost‐effectiveness.

**Tweetable abstract:**

Oral misoprostol less costly and more effective than Foley catheter for labour induction in hypertension.

## Tweetable abstract

Oral misoprostol less costly and more effective than Foley catheter for labour induction in hypertension.

## Introduction

Hypertensive disorders, including pre‐eclampsia, are the most common medical complication of pregnancy, accounting for ~14% of the estimated 303 000 global annual maternal deaths.^1^, ^2^ A great deal of this burden is experienced in developing countries, where the incidence of pre‐eclampsia is increased considerably.^3^, ^4^

Timely delivery, preferably by vaginal route, remains the only definitive cure for pre‐eclampsia and is therefore vital to achieve favourable maternal and neonatal outcomes. Hence, the induction of labour is a critical intervention in the management of hypertension in pregnancy. Two low‐cost methods, low‐dose oral misoprostol and the Foley balloon catheter, have been previously recommended for the induction of labour within low‐resource settings, but are yet to be directly compared.^5^

The prostaglandin E1 analogue oral misoprostol is a highly effective induction agent;^6^ however, it carries a uterine hyperstimulation rate of 5–10%,^7^ potentially resulting in hypoxic damage to the fetus. Although evidence from low‐resource settings is scant, studies conducted in developed health economies suggest Foley balloon catheterisation may be equally effective as oral misoprostol for the induction of labour, with lower rates of uterine hyperstimulation,^8^, ^9^, ^10^ but also a slower speed of induction and increased requirement for caesarean section.^8^ Induction with the Foley balloon catheter may therefore result in a reduction of risk to the fetus, but with the caveat of a slower labour and an increased use of oxytocin. Because in many low‐resource settings, oxytocin is administered under gravity alone (using drip counters), it is possible that any neonatal benefits from Foley balloon induction may be outweighed by the complications of overdosage with oxytocin.

To date, the sum of available evidence suggests both methods are promising; however, the relative cost‐effectiveness of these methods for induction of labour in women with gestational hypertension remains unknown in low‐resource settings. We conducted a cost‐consequence analysis of a previously reported multicentre randomised controlled trial (RCT),^11^ comparing oral misoprostol with Foley balloon induction in women with gestational hypertension. We compared the respective efficacy, healthcare resource utilisation, and adverse event profile of these therapeutic indications for the induction of labour among those with gestational hypertension in a low‐resource setting.

## Materials & methods

### Study design & Participants

We undertook a cost‐consequence analysis of a previously reported multicentre, parallel, open‐label randomised trial at two public hospitals in Nagpur, India, between December 2013 and June 2015. The study was approved by the Research Ethics Committees at Government Medical College, and the University of Liverpool. As required by the Drug Controller General of India, women provided both written and video‐recorded oral consent. The trial is registered with the clinical trials registry ClinicalTrials.gov: NCT01801410.

The trial protocol is published elsewhere.^12^ In short, however, women requiring delivery for hypertension or pre‐eclampsia were randomised to either cervical ripening with transcervical Foley catheter or 25 mcg oral misoprostol tablets given every 2 hours. Only women ≥18 years of age with continuing pregnancies and a live fetus, in whom the decision had been made to induce vaginal delivery because of pre‐eclampsia or hypertension, were eligible to participate. Women unable to give informed consent and those with a prior caesarean delivery, multiple pregnancy, ruptured membranes, clinically diagnosed chorioamnionitis, or a history of allergy to misoprostol were ineligible for the trial.

### Randomisation and masking

Women were informed about the study by their doctor when the need for induction of labour occurred, and enrolled by research staff on the labour ward on the day of induction. After informed consent, a sequentially numbered, sealed, opaque envelope containing the participant's group assignment in a 1:1 ratio was opened by research staff. The randomisation was stratified by centre and used randomly assigned block sizes of 4, 6, and 8. Due to differences in administrative method between the two interventions, the masking of intervention allocation would have been very difficult and was therefore not performed.

### Procedures/Interventions

Prior to randomisation, the resident doctor performed a digital examination, to establish a baseline Bishop score and cervical dilation. Women randomised to the Foley catheter arm underwent induction using a transcervical Foley catheter (silicone, size 18F with 30‐ml balloon). The catheter remained in place until it was expelled when active labour started, or alternatively, until 12 hours had elapsed, in which case an artificial rupture of membranes (ARM) was performed, and an oxytocin infusion commenced. Similarly, if the Foley catheter fell out within 12 hours, the membranes were ruptured and an oxytocin infusion commenced.

Women assigned to the misoprostol group were induced using oral misoprostol tablets (Cipla Misoprost 25 mcg), every 2 hours for a maximum of 12 doses (24 hours) or until active labour commenced. In primigravida women, if contractions had not commenced after 2 doses, the dosage could be increased to 50 mcg every 2 hours. Once in labour (defined as regular painful contractions with a cervical dilation of at least 4 cm), no more misoprostol was used and artificial membrane rupture and/or oxytocin infusion was used as clinically indicated. In both arms, if labour had not commenced after 24 hours, the case was considered a ‘failed induction’ and the decision on further management was made by the clinical team.

For women in both groups, oxytocin was administered with a regular drip infusion set, monitored by counting the number of drops per minute. One unit of oxytocin was injected into 500 ml of Ringer's lactate, started at a rate of 2 mU/minute (15 drops/minute), and increased every 30 minutes by 2 mU/min until there were three–four contractions in ten minutes. All women were monitored by the research staff on a one‐to‐one basis. Participants with severe hypertension received magnesium sulphate and antihypertensives both before and after randomisation as per the hospital protocol.

### Outcomes

The primary clinical outcome of the clinical trial was the achievement of vaginal delivery within 24 hours of induction. As such, our cost‐consequence analysis considered the comparative achievement of vaginal delivery, delivery (by any method) within 24 hours of induction, and a composite measure of vaginal delivery within 24 hours of commencing induction. We also report the comparative ‘costs per successful vaginal delivery within 24 hours of induction’, from the perspective of the Indian healthcare system. Although the study was neither designed nor statistically powered for subgroup analyses, exploratory subgroup analyses were used to highlight potentially important differences in the cost‐effectiveness of the two treatments, which could be attributed to differences in observable patient characteristics.

Healthcare expenditure was estimated by multiplying the observed utilisation of healthcare resources, as recorded at the patient's bedside by trial administrators, by associated unit costs obtained from the finance department of Government Medical College, Nagpur, India. Because unit costs were obtained in Indian rupees (INR) for the financial year of 2014/2015, costs were inflated using the consumer price index, and then converted into US dollars (USD) using a purchasing power parity‐adjusted exchange rate of 17.22INR to 1USD as estimated by the World Bank.^13^ Because data were non‐normally distributed, 95% confidence intervals for treatment costs were imputed using 5,000 nonparametric gamma bootstrap simulations, followed by the percentile method to define lower and upper confidence limits. Sampling distributions were derived from the observed mean and standard deviation of each cost component (delivery, induction, inpatient, neonatal), for each treatment group. All unit costs are reported in Table S1. We additionally assessed the acceptability of each induction method by asking participants about (1) self‐reported pain experienced, (2) acceptability with the amount of time taken, and (3) whether participants would use the same method for induction again.

### Statistical analysis

We used summary statistics to describe the characteristics of the trial groups at baseline. Categorical variables were summarised by frequency and percentage, while continuous variables were reported as mean and standard deviation (SD). We analysed data for the primary economic outcome from a modified intention‐to‐treat (ITT) perspective, including all randomly assigned participants, except for those in whom primary outcome data were missing, due to withdrawal from the trial postrandomisation. Normally distributed continuous variables were compared using the Student t‐test.

The sample size was estimated *a priori*, assuming a vaginal delivery rate of 41% with the Foley catheter, based on previously published data using identical induction protocols and outcomes to this study.^14^, ^15^, ^16^ Full details of the sample size calculation, in addition to data concerning the occurrence of adverse events, which bore no clear and translatable cost to the healthcare providers (e.g. headache, maternal vomiting, and meconium‐stained liquor), are reported elsewhere.^11^

## Results

### Recruitment & Clinical efficacy

Between December 2013 and June 2015, 2,412 women were assessed for eligibility, with 602 women included in the trial (Figure S1). For a single patient, primary outcome data were missing for the primary outcome, and for this reason, this patient was excluded from the analysis, resulting in a total of 601 participants in a modified intention‐to‐treat analysis. Baseline characteristics were similar for the two groups, as shown in Table 1.

**Table 1:** Baseline characteristics of study groups

Measure	Foley catheter (*n* = 300)	Misoprostol (*n* = 302)
**Study site**		
GMC, *n* (%)	150 (50.0)	151 (50.0)
Daga, *n* (%)	150 (50.0)	151 (50.0)
**Background**		
Woman's age, mean (SD) [range]	24.0 (3.5) [18–42]	23.7 (3.1) [18–37]
Mother's education		
No formal education, *n* (%)	5 (1.7)	2 (0.7)
Primary, *n* (%)	86 (28.7)	112 (37.1)
Secondary, *n* (%)	149 (49.7)	131 (43.3)
University, *n* (%)	60 (20.1)	57 (19.0)
**Medical history**		
Nulliparous (no previous pregnancies >28 weeks), *n* (%)	247 (82.3)	236 (78.1)
Previous hypertension in pregnancy: *n* (%)	8 (2.7)	16 (5.3)
Previous stillbirth, *n* (%)	1 (0.3)	5 (1.7)
Pre‐existing diabetes/renal or liver disease, *n* (%)	0	0
Pre‐existing chronic hypertension, *n* (%)	0	1 (0.3)
**State at recruitment**		
Gestational age (best estimate in weeks), mean (SD) [range]	38.2 (2.2) [29–42]	38.1 (2.1) [29–41]
Estimate made by ultrasound at <20 weeks, *n* (%)	131 (43.7)	127 (42.1)
Systolic BP (mm/Hg), mean (SD) [range]	142.2 (11.3) [104–180]	142.8 (12.5) [102–190]
Diastolic BP (mm/Hg), mean (SD) [range]	95.0 (8.3) [60–130]	94.7 (8.3) [66–120]
Proteinuria at enrolment		
Nil or trace, *n* (%)	156 (52.0)	162 (53.7)
+1 / +2, *n* (%)	122 (40.6)	121 (40.0)
+3 / +4, *n* (%)	22 (7.4)	19 (6.3)
Hypertensive symptoms at enrolment: *n* (%)	64 (21.3)	58 (19.2)
Woman received MgSO4 in last 12 hours, *n* (%)	45 (15.0)	42 (13.9)
Woman currently on antihypertensives, *n* (%)	292 (97.3)	289 (95.7)

Those receiving oral misoprostol 25 mcg demonstrated greater achievement of the primary clinical outcome of the trial, with 57% [95% CI (51.4–62.5%)], as opposed to 47% [95% CI (41.5–52.8%)] in the Foley group achieving a vaginal delivery within 24 hours of induction (*P* = 0.0162). Vaginal delivery was observed in 59.3% and 49.8% of misoprostol and Foley patients, respectively (*P* = 0.0210), while 92.5% of misoprostol and 89.3% of Foley patients delivered within 24 hours of induction (*P* = 0.1913).

### Determinants of costs, and treatment acceptability

Misoprostol patients incurred a mean treatment cost of $117.5 during their hospital episode [95% CI $49.73–$202.73], a 14.9%, or $20.6 reduction when compared to those receiving Foley catheterisation, at $138.1 per patient [95% CI $56.83–$246.66, *P* < 0.0001]. Those randomised to the Foley group incurred a mean induction cost of $26.4 per patient [95% CI ($8.92–$50.91)], compared to $15.7 per patient [95% CI ($1.26–$39.67)] in those receiving oral misoprostol. Most of this difference was attributable to a significantly higher utilisation of oxytocin in the Foley group (81.6% vs. 52%), an increased duration of oxytocin administration (5.9 vs 2.5 hours per patient (*P* < 0.0001)), and an increased use of artificial rupture of membranes (77.2% vs. 60.7%, *P* = 0.001).

Delivery‐related healthcare expenditure was reduced, on average, by $2.3 (95% CI $1.34–$3.79) per patient in those receiving oral misoprostol. This saving was attributable, in the majority, to the significant reduction in caesarean‐section rate (50.3 vs. 41.1%, *P* = 0.025), and spinal anaesthesia (50% vs. 41.1%, *P* = 0.0275) for oral misoprostol patients, as demonstrated in Table 2.

**Table 2:** Utilisation rates and determinants of cost difference between Foley catheterisation and oral misoprostol 25 mcg

	Foley catheterisation (*n* = 299)	Cost per patient ($)	Oral misoprostol (*n* = 302)	Cost per patient ($)	*P*‐value
**Induction‐related determinants of costs**					
Antihypertensives (mg per person)					
Nifedipine	8.96	$0.08	6.6	$0.06	0.1712
Aldomet	340.3	$0.28	351.8	$0.29	0.7169
Labetalol	14.7	$0.15	16.9	$0.17	0.5996
Antibiotics (mg per person)					
Cifran IV	4.7	$0.03	0	$0.00	0.0346
Metronidazole IV	0	$0.00	2.7	$0.10	0.1576
Taxim IV	33.4	$0.95	33.1	$0.06	0.9853
Analgesics (mg per person)					
Paracetamol	13.4	$0.01	11.6	$0.01	0.7792
Other					
MgSO4 (gm per person)^a^	1.74	$1.47	1.69	$1.41	0.8972
Oxytocin (minutes of infusion per person)	432.3	$9.08	297	$4.12	0.000
ARM^b^	193 (77.2%)	$8.21	153 (60.7%)	$6.38	0.001
**Delivery‐related determinants of costs**					
Caesarean	150 (50.2%)	$15.79	124 (41.1%)	$12.93	0.025
Spinal anaesthesia	149 (49.8%)	$15.69	124 (41.1%)	$12.93	0.0308
Local anaesthesia	94 (31.4%)	$3.98	114 (37.7%)	$4.59	0.1968
Episiotomy^c^	96 (64.4%)	$4.05	118 (65.9%)	$4.88	0.0891
**Inpatient determinants of costs**					
Time (hours) from randomisation to induction	0.56	$0.19	0.16	$0.05	0.0001
Time (hours) from induction to delivery	14.35	$4.90	12.85	$4.38	0.0008
Time (hours) from delivery to discharge	136.96	$46.74	125.45	$42.81	0.1503
Total time as inpatient (hours)	151.86	$51.82	138.46	$47.25	0.0432
**Neonatal determinants of costs**					
Ventilation (min)	50.05	$0.44	26.03	$0.23	0.736
Oxygen administration (min)	82.35	$0.36	86.62	$0.38	0.4165
NICU stay (min)	491.15	$4.35	548.24	$4.80	0.8087

a Including costs of fluids and intracatheters to administer MgSO4

b Out of those with rupture time recorded

c Out of 149 vaginal deliveries in Foley group vs. 179 vaginal deliveries in misoprostol group

Those undergoing Foley catheterisation also exhibited higher inpatient costs than those receiving oral misoprostol. The time between randomisation and commencing induction was almost four times greater for Foley patients (0.56 to 0.16 hours, *P* = 0.0004), while the time from induction to delivery was reduced by approximately 90 minutes for those receiving oral misoprostol (14.35 vs. 12.85 hours, *P* = 0.0094). Additionally, in the postpartum period, patients receiving oral misoprostol spent on average 11.4 hours fewer in hospital prior to discharge (136.96 vs. 125.45 hours, *P* = 0.0792). The costs of neonatal care were almost equivalent in both groups, with a $3.3 saving (95% CI (‐)$1.06–$7.67) in favour of Foley catheterisation. Most women in both groups found their assigned method of induction, and the duration of the induction, to be acceptable, and the pain they experienced to be either slight or moderate (Table 3). More women in the misoprostol group (82.8%) than the Foley catheter group (72%) would use the same method in the future should they require another induction (Table 3; *P* = 0.006).

**Table 3:** Maternal outcomes for those receiving Foley catheterisation and oral misoprostol 25 mcg

	Foley catheter (*n* = 300)	Oral misoprostol (*n* = 302)	Mean difference (95% CI)	*P*‐value
Vaginal birth within 24 hours	141 (47%)	172 (57%)	10.0% (−2.0 to 17.9)	0.0136
Delivered within 24 hours	268 (89.3%)	279 (92.4%)	3.1% (−1.5 to 7.6)	0.194
Vaginal birth	149 (49.7%)	178 (58.9%)	9.3% (1.3 to 17.2)	0.0212
**Mode of birth**				
Spontaneous vaginal birth	146 (48.7%)	176 (58.3%)	9.6% (1.7 to 17.5)	0.0194
Forceps or vacuum birth	3 (1%)	2 (0.7%)	−0.3% (−1.8 to 1.1)	
Caesarean section	151 (50.3%)	124 (41.1%)	−9.2% (−17.2 to −1.3)	0.025
Oxytocin required	244 (81.6%)	157 (52%)	−29.6% (−36.8 to −22.5)	<0.0001
Hours of oxytocin	5.9	2.5	3.4 (2.7 to 4.1)	<0.0001
Total time spent in hospital	151.6	138.4	13.2 (−2.9 to 29.2)	0.0537
Randomisation to induction	0.56	0.16	0.4 (0.17 to 0.63)	0.0004
Induction to delivery	14.3	12.9	1.4 (0.2 to 2.6)	0.0094
Delivery to discharge	136.8	125.4	11.4 (−4.4 to 27.1)	0.0792
**Analgesia**				
Spinal anaesthesia	150 (50%)	124 (41.1%)	−8.9% (−16.9 to −1.0)	0.0275
Local anaesthesia	94 (31.3%)	114 (37.7%)	6.4% (−1.2 to 14.0)	0.097
**Complications of labour and birth**				
Uterine hyperstimulation	1 (0.3%)	2 (0.7%)	0.3% (−0.8 to 1.5)	0.566
Fetal heart rate abnormality	17 (5.7%)	12 (4%)	−1.7% (−5.1 to 1.7)	0.332
Diagnosis of postpartum haemorrhage	2 (0.7%)	2 (0.7%)	0 (−1.3 to 1.3)	0.995
Blood products after trial entry	5 (1.7%)	1 (1.3%)	−1.3% (−2.9 to 0·3)	0.099
Severe hypertension	21 (7%)	23 (7.6%)	0.6% (−3.5 to 4.8)	0.772
Any form of complication	44 (14.7%)	37 (12.3%)	−2.4% (−7.9 to 3.0)	0.385
**Adverse effects during induction**				
Mild diarrhoea	2 (0.7%)	7 (2.3%)	1.7% (−0.3 to 3.6)	0.094
**Amount of pain experienced**				
None/slight	91 (30.3%)	86 (28.5%)		
Moderate	145 (48.3%)	152 (50.3%)		
High/extreme	64 (21.3%)	64 (21.2%)		
**Acceptability of amount of time taken**				
Very acceptable	49 (16.4%)	52 (17.2%)		
Acceptable	129 (43.1%)	145 (48.0%)		
Neutral	81 (27.1%)	75 (24.8%)		
Unacceptable	35 (11.7%)	26 (8.6%)		
Very unacceptable	5 (1.7%)	4 (1.3%)		
**Would use same method again?**				
Yes	216 (72%)	250 (82.8%)		
No	59 (19.7%)	35 (11.6%)		0.006
No preference	25 (8.3%)	17 (6%)		

### Maternal and neonatal outcomes

No significant difference in adverse events was observed. Uterine hyperstimulation occurred in 0.3% and 0.7% of the Foley and misoprostol groups, respectively (*P* = 0.566). Similarly, rates of fetal heart rate abnormality (5.7% vs. 4.0%), severe hypertension (7.0% vs. 7.6%), postpartum haemorrhage (0.7% vs. 0.7%), and use of blood products after trial entry (1.7% vs. 0.3%) were not statistically different. Two babies (1%) were stillborn to women induced with the Foley catheter, and nine babies (1%) died in total, three in the Foley group (all due to prematurity) and six in the misoprostol group (three due to prematurity, one from prematurity plus intrauterine growth restriction, one from intrauterine growth restriction alone, and one from asphyxia). The causes of death did not differ significantly between the two groups. Neonatal morbidity, as judged by Apgar scores, asphyxiation, admission to special care units, ventilation, and oxygen administration rates were similar in both groups, and further details of the adverse event profile of each treatment are provided in Tables 3 and 4.

**Table 4:** Neonatal outcomes for those receiving Foley catheterisation and oral misoprostol 25 mcg

	Foley catheter (*n* = 300)	Oral misoprostol (*n* = 302)	Mean difference (95% CI)	*P*‐value
**Outcome of birth**				
Live birth	298 (99.3%)	302 (100%)	0.70%	
Stillbirth	2 (0.7%)	0		
**Birthweight (g)**				
Mean (SD)	2612 (464)	2616 (490)	4 (−72 to 80)	0.918
Median (Range)	2600 (1000–3830)	2600 (750–3800)		
**Apgar score at 1 minute**				
<7	10 (3.4%)	12 (4%)	0.6% (−2.4 to 3.6)	0.687
>7	288 (96.6%)	290 (96%)		
**Apgar score at 5 minutes**				
<7	1 (0.3)	6 (2%)	1.7% (−0.1 to 3.4)	0.058
>7	297 (99.7%)	296 (98%)		
**Apgar score at 10 minutes**				
<7	0	5 (1.7%)	1.70%	0.431
>7	298 (100%)	297 (98%)		
**Other neonatal outcomes**				
Neonatal death	3 (1%)	6 (2%)	1.0% (−1.04 to 2.97)	0.322
Baby admitted to special care nursery	19 (6.4%)	28 (9.3%)	2.9% (−1.4 to 7.2)	0.186
Baby given oxygen	33 (11.1%)	42 (13.9%)	2.8 (−2.5 to 8.1)	0.293
Baby ventilated	4 (1.3%)	4 (1.3%)	0 (−1.9 to 1.8)	0.985
Sarnat score completed	19 (6.3%)	29 (9.6%)	3.3% (−1.0 to 7.6)	0.138
Normal	13 (68.4%)	20 (69%)		
Moderate	6 (31.6%)	8 (27.6%)		
Severe	0	1 (3.4%)		

### Sensitivity analysis

Following probabilistic sensitivity analysis, oral misoprostol was cost‐saving in 63% of 5,000 bootstrap replications. Oral misoprostol also achieved superior rates of delivery within 24 hours of induction, vaginal delivery, and vaginal delivery within 24 hours of induction in 90.7%, 98.7%, and 99.4% of bootstrap simulations. Based on univariate threshold analysis, the unit price of oral misoprostol 25 mcg could feasibly increase 31‐fold from $0.24 to $7.50 per 25 mcg tablet and still remain weakly dominant over Foley catheterisation, resulting in equivalent costs and improved rates of induction within 24 hours of labour.

### Subgroup analyses

As expected, healthcare expenditure per completed treatment episode increased with the extent of prematurity, as shown in Table S2. Oral misoprostol demonstrated resource savings over Foley catheterisation at all gestational ages, in addition to demonstrating improved effectiveness, the extent of which increased with the extent of prematurity. For those with a Bishop's score of ≥3, oral misoprostol resulted in a $15.3 per patient reduction in treatment costs and a 13% improvement in vaginal delivery within 24 hours of induction (52% vs. 58.8%, *P* = 0.12). For those with a Bishop's score of <3, almost twice as many women delivered vaginally within 24 hours in the oral misoprostol cohort (45% vs. 22.7%) (*P* = 0.03), while healthcare expenditure was also reduced by $37.6 per patient.

## Discussion

### Main findings

The results of this multicentre randomised trial, performed in two hospitals within the Maharashtra Province of India, demonstrate that for the induction of hypertensive women in low‐resource settings, low‐dose oral misoprostol 25 mcg is both more clinically effective and less resource‐intensive than transcervical Foley catheterisation. 57% [95% CI (51.4–62.5%)] of our oral misoprostol group, as opposed to 47% [95% CI (41.5–52.8%)] in the Foley group, achieved a vaginal delivery within 24 hours of induction (*P* = 0.0162), while mean treatment costs equalled $138.10 per patient [95% CI $127.06–$146.28] in the Foley group, reducing by 14.9% to $117.51 per patient [95% CI $111.06–$123.45] in the oral misoprostol group. This $20.6 saving per patient could have provided a 40‐hour stay in ICU, or 77 hours of oxygen administration in this low‐resource setting. Sensitivity analysis demonstrated a 63% probability of oral misoprostol being cost‐saving over Foley catheterisation, and a 90.7%, 98.7%, and 99.4% probability of achieving superior rates of delivery within 24 hours of induction, vaginal delivery, and vaginal delivery within 24 hours of induction, respectively.

### Strengths & limitations

A key strength of this study is that to the best of our collective knowledge, it is the first of its kind to demonstrate the relative cost‐effectiveness and budget impact of these two treatments for the induction of labour in hypertensive women. Additionally, the study relied upon internally collected financial data concerning real‐world purchasing and reimbursement costs for the hospitals involved, while all observations concerning patient‐level resource use were collected at the patient's bedside via trial administrators, resulting in considerable precision.

The limitations of this study primarily concern the real‐world validity of several assumptions. First, outside of trial conditions, it is unclear whether midwives would have the capacity to continuously provide oral misoprostol at optimal two‐hourly intervals. As such, the efficacy of oral misoprostol demonstrated within this trial may be greater than that which we would expect to observe in the real world. Second, the financial costs of staff time, whether nurse, junior doctor, or consultant, were accounted for on an equal basis, due to the unavailability of data concerning individual staff salaries. While oral misoprostol can be administered by most staff members, a greater skill level is necessary to insert a Foley catheter, suggesting that the costs of Foley insertion were possibly underestimated during this analysis. Third, hospitals vary hugely in their approach to intrapartum protocols. The oral misoprostol and Foley catheter protocols described in this study are based on previous studies, guidelines, and expert advice. However, they are not the definitive versions, and the costs (and clinical outcomes) could vary considerably with even small variations in indication, oxytocin use, or staff supervision. Settings both within India and internationally will also vary in their rates of caesarean section and costs of neonatal care, and these could have marked effects on the cost‐effectiveness. The results of this study can only therefore be viewed as an indication of what happens with a typical protocol and hospital setting. Of particular note is the absence of intrapartum continuous electronic monitoring and electronic oxytocin pumps. This increases its applicability and generalisability to other low‐resource settings without these technologies, but limits its applicability to settings where these technologies are more readily available.

### Interpretation in the light of other evidence

The induction of labour is a critical intervention in the management of hypertension in pregnancy. Two low‐cost methods, low‐dose oral misoprostol and the Foley balloon catheter, have been previously recommended for the induction of labour within low‐resource settings, with both found to have advantages over other induction methods in systematic reviews,^6^, ^7^, ^8^, ^10^ but until recently, they had never been directly compared.

Due to a lack of effect on uterine contractions during the cervical ripening phase,^8^, ^10^ Foley catheterisation has been shown to result in safe but slow labours, which avoid the dangers of hyperstimulation, but may result in increased requirement for both caesarean section^8^ and additional need for labour augmentation with oxytocin. This was observed within our study, with 57% of misoprostol and 47% of Foley patients achieving a successful induction. As a result, over 80% of our Foley cohort required additional uterine stimulation with oxytocin in comparison with just 52% of the misoprostol cohort, a finding synonymous with existing literature.^10^ Furthermore, among those who did require oxytocin infusion, the duration of infusion also increased by 57% for those in the Foley group (432.3 vs. 297 minutes). This resulted in a greater use of limited healthcare resources during the induction interval. Furthermore, because in many low‐resource settings, oxytocin is administered under gravity alone, without the safeguards of electronic infusion control, any reduction in oxytocin usage may not only reduce health service costs, but also improve maternal safety, with the risks associated with oxytocin overdosage falling.

Additionally, given the increased susceptibility for failed inductions, literature collected in Western settings has demonstrated that caesarean‐section rates may be higher in those induced with the Foley balloon catheter, when compared to other induction methods,^8^, ^17^ and the results of this study, performed in a low‐resource setting, corroborate this finding. Those receiving the Foley catheter experienced an 18.1% increase in caesarean‐section rates relative to those receiving oral misoprostol, suggesting that not only is the use of Foley catheterisation in this setting likely to result in an escalation of risk to patients, given considerations of infection control and the general risks of anaesthesia, but also likely to increase pressures on nursing staff, hospital beds, and highly skilled theatre technicians, all of which are likely already in both high demand and short supply.

Given the high prevalence of pre‐eclampsia,^1^, ^2^, ^18^ in addition to low levels of investment in publicly funded health care in India (1.3% of GDP),^19^, ^20^ the discovery that oral misoprostol results in both improvements in clinical outcomes and reductions in healthcare expenditure is an important finding. The $5,611.4222 difference in total healthcare expenditure between the two arms of this trial over the study period could have otherwise provided 89 caesarean sections, 445 days in a special care baby unit, or 3,563 bags of saline solution. As such, the opportunity for similar savings to be achieved on a larger scale, which could then be used to promote health where unmet clinical need is greatest, could have considerable impact.

Further research should aim to determine whether the results observed in this province of India are generalisable to other provinces or low‐resource settings, and whether widening the inclusion criteria to better reflect routine clinical practice, including those with a prior C‐section, would change the study conclusions. There are a wide variety of induction methods available, but this study relates only to these two specific methods. For example, some practitioners are using the Foley catheter at the same time as low‐dose misoprostol to improve outcomes, and this also deserves further research. Widening the perspective of the analysis beyond solely health‐service‐related outcomes would also provide valuable insights as to the societal impact of each treatment indication, particularly with respect to time away from work, impact on ability to perform household duties, and the financial costs of birthing partners requiring accommodation for the duration of hospital stay.

## Conclusion

The results of this study suggest that when compared to Foley catheterisation for the induction of high‐risk hypertensive women, oral misoprostol improves rates of vaginal delivery, delivery within 24 hours of induction, and vaginal delivery within 24 hours of induction and may also reduce costs. Additional research performed in other low‐resource settings is essential to determine the definitive cost‐effectiveness of these two treatments.

### Disclosure of interests

Full disclosure of interests available to view online as supporting information.

### Contribution to authorship

ADW had the original idea for the study and is guarantor for the study. The idea was then developed into a formal grant application with SM, BW, HB, ZA, BF, TE, and AH. SM led the study team in India, with VK and JM as local principle investigators for the study sites, and MT joined to provide academic neonatal support. SM, HB, BF, SL, BW, and ADW formed the trial management team with input from other co‐investigators as required. HB was the study monitor. SL performed all data cleaning and formatting, and planned and conducted the economic analysis, with PG performing statistical analyses. SL wrote the first and subsequent drafts of the economic analysis manuscript. All authors reviewed and accepted the manuscript prior to submission.

### Details of ethics approval

The study was approved by the Research Ethics Committees at Government Medical College (3rd September 2012, ref # 320/12), the University of Liverpool (1st October 2012) and the Indian Council of Medical Research (9th October 2013, ref # 5/7/948/13‐RCH). As required by the Drug Controller General of India, women provided both written and video‐recorded oral consent. The trial is registered with the clinical trials registry ClinicalTrials.gov: NCT01801410.

### Funding

This trial was funded by a grant to the University of Liverpool from the DFID/MRC/Wellcome Trust through the Joint Global Health Trials Scheme.

## Supporting information


**Figure S1.** CONSORT flow chart for the study.

**Table S1.** Unit costs of healthcare resource utilisation.

**Table S2.** Comparison of healthcare costs for Foley catheterisation and oral misoprostol 25 mcg.■

## References

[1] Say L , Chou D , Gemmill A , Tunçalp Ö , Moller AB , Daniels J , et al. Global causes of maternal death: a WHO systematic analysis. Lancet Glob Health 2014;2:e323–33. 25103301 10.1016/S2214-109X(14)70227-X

[2] Alkema L , Chou D , Hogan D , Zhang S , Moller AB , Gemmill A , et al. Global, regional, and national levels and trends in maternal mortality between 1990 and 2015, with scenario‐based projections to 2030: a systematic analysis by the UN Maternal Mortality Estimation Inter‐Agency Group. Lancet 2015;387:462–74. 26584737 10.1016/S0140-6736(15)00838-7 PMC5515236

[3] Altman D , Carroli G , Duley L , Farrell B , Moodley J , Neilson J , et al. Do women with pre‐eclampsia, and their babies, benefit from magnesium sulphate? The Magpie Trial: a randomised placebo‐controlled trial. Lancet 2002;359:1877–90. 12057549 10.1016/s0140-6736(02)08778-0

[4] Aabidha PM , Cherian AG , Paul E , Helan J . Maternal and fetal outcome in pre‐eclampsia in a secondary care hospital in South India. J Family Med Prim Care 2015;4:257–60. 25949977 10.4103/2249-4863.154669 PMC4408711

[5] WHO . WHO Recommendations for Induction of Labour. Geneva, Switzerland: World Health Organisation, 2011. 23586118

[6] Alfirevic Z , Keeney E , Dowswell T , Welton NJ , Dias S , Jones LV , et al. Labour induction with prostaglandins: a systematic review and network meta‐analysis. BMJ 2015;350:h217. 25656228 10.1136/bmj.h217 PMC4353287

[7] Alfirevic Z , Aflaifel N , Weeks A . Oral misoprostol for induction of labour. Cochrane Database Syst Rev 2014;6:CD001338. 10.1002/14651858.CD001338.pub3 PMC6513439 24924489

[8] Chen W , Xue J , Peprah MK , Wen SW , Walker M , Gao Y , et al. A systematic review and network meta‐analysis comparing the use of Foley catheters, misoprostol, and dinoprostone for cervical ripening in the induction of labour. BJOG 2016;123:346–54. 26538408 10.1111/1471-0528.13456

[9] Jozwiak M , Oude Rengerink K , Benthem M , van Beek E , Dijksterhuis MG , de Graaf IM , et al. Foley catheter versus vaginal prostaglandin E2 gel for induction of labour at term (PROBAAT trial): an open‐label, randomised controlled trial. Lancet 2011;378:2095–103. 22030144 10.1016/S0140-6736(11)61484-0

[10] Jozwiak M , Bloemenkamp KW , Kelly AJ , Mol BW , Irion O , Boulvain M . Mechanical methods for induction of labour. Cochrane Database Syst Rev 2012;3:CD001233. 10.1002/14651858.CD001233.pub2 22419277

[11] Mundle S , Bracken H , Khedikar V , Mulik J , Faragher B , Easterling T , et al. Foley catheterisation versus oral misoprostol for induction of labour in hypertensive women in India (INFORM): a multicentre, open‐label, randomised controlled trial. Lancet 2017;10095:669–80. 10.1016/S0140-6736(17)31367-3 28668289

[12] Bracken H , Mundle S , Faragher B , Easterling T , Haycox A , Turner M , et al. Induction of labour in pre‐eclamptic women: a randomised trial comparing the Foley balloon catheter with oral misoprostol. BMC Pregnancy Childbirth 2014;14:308. 25193157 10.1186/1471-2393-14-308 PMC4261642

[13] The World Bank International Comparison Program Database: PPP conversion factor. [http://data.worldbank.org/indicator/PA.NUS.PPP] Accessed 6th February 2017.

[14] Pennell CE , Henderson JJ , O'Neill MJ , McCleery S , Doherty DA , Dickinson JE . Induction of labour in nulliparous women with an unfavourable cervix: a randomised controlled trial comparing double and single balloon catheters and PGE2 gel. BJOG 2009;116:1443. 19656148 10.1111/j.1471-0528.2009.02279.x

[15] Owolabi AT , Kuti O , Ogunlola IO . Randomised trial of intravaginal misoprostol and intracervical Foley catheter for cervical ripening and induction of labour. J Obstet Gynaecol 2005;25:565–8. 16234141 10.1080/01443610500231450

[16] Yuen PM , Pang HYY , Chung T , Chang A . Cervical ripening before induction of labour in patients with an unfavourable cervix: a comparative randomized study of the Atad ripener device, prostaglandin E2 vaginal pessary, and prostaglandin E2 intracervical gel. Aus NZ J Obstet Gynecol 1996;36:291–5. 10.1111/j.1479-828x.1996.tb02713.x 8883753

[17] Ten Eikelder ML , Oude Rengerink K , Jozwiak M , de Leeuw JW , de Graaf IM , van Pampus MG . Induction of labour at term with oral misoprostol versus a Foley catheter (PROBAAT‐II): a multicentre randomised controlled non‐inferiority trial. Lancet 2016;387:1619–28. 26850983 10.1016/S0140-6736(16)00084-2

[18] Dolea C , AbouZahr C . Global Burden of Hypertensive Disorders of Pregnancy in the Year 2000. Geneva, Switzerland: World Health Organization, 2000.

[19] Narain JP . Public health challenges in India: seizing the opportunities. Indian J Community Med 2016;41:85–8. 27051080 10.4103/0970-0218.177507 PMC4799645

[20] Balarajan Y , Selvaraj S , Subramanian SV . Health care and equity in India. Lancet 2011;377:505–15. 21227492 10.1016/S0140-6736(10)61894-6 PMC3093249

