# Supplementary figures and images for: Foley catheter vs. oral misoprostol to induce labour among hypertensive women in India: a cost‐consequence analysis alongside a clinical trial
Source: BJOG. 2018 Jun 22;125(13):1734–42. doi: 10.1111/1471-0528.15285 (PMC6282740; doi:10.1111/1471-0528.15285)

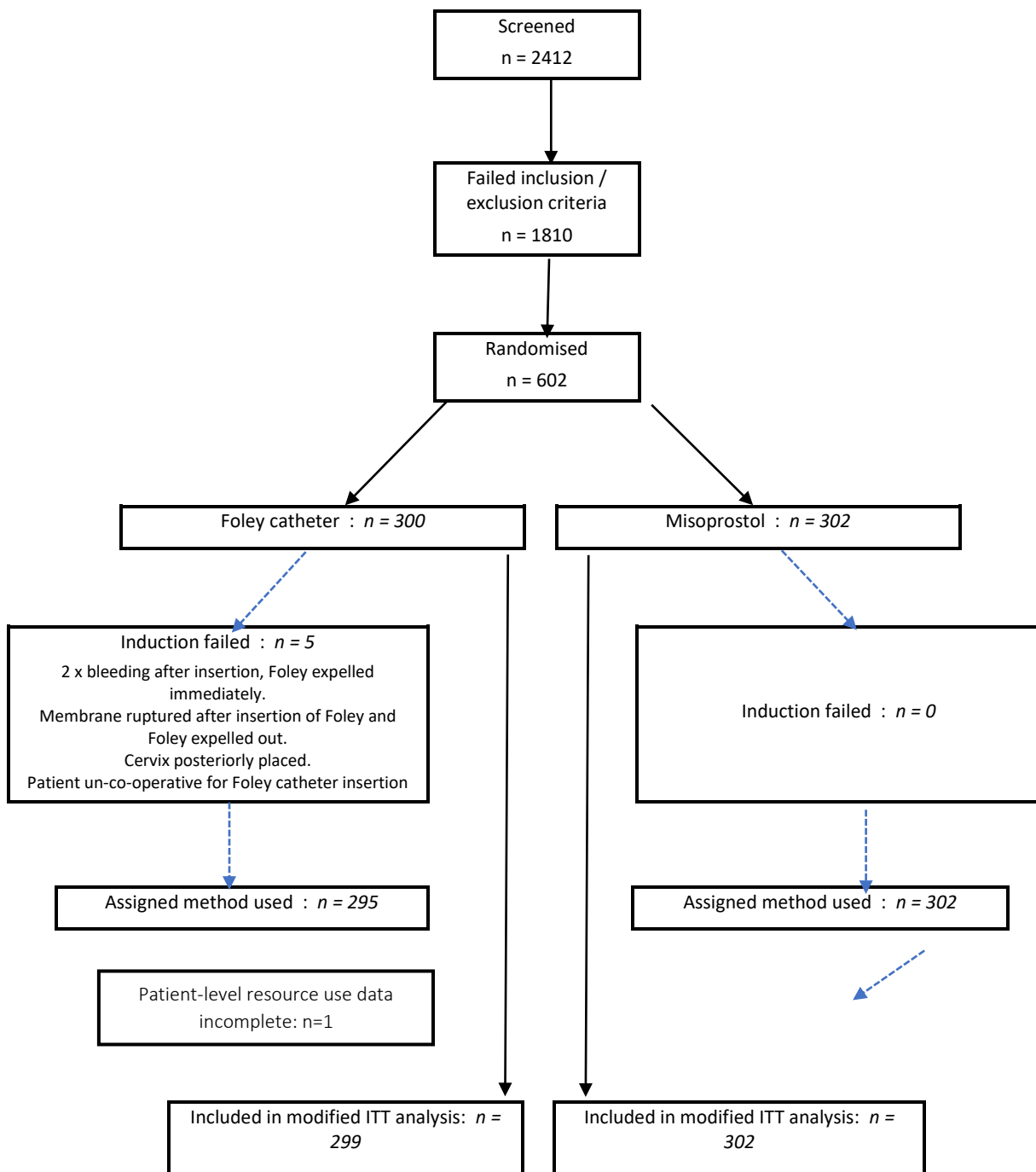

**Figure S1.** CONSORT flow chart for study

Supplement: Supplementary file 1 — Figure S1. CONSORT flow chart for the study. [file BJO-125-1734-s001.pdf]
