# Supplementary material for: Foley catheter vs. oral misoprostol to induce labour among hypertensive women in India: a cost‐consequence analysis alongside a clinical trial
Source: BJOG. 2018 Jun 22;125(13):1734–42. doi: 10.1111/1471-0528.15285 (PMC6282740; doi:10.1111/1471-0528.15285)
Supplement: Supplementary file 2 — Table S1. Unit costs of healthcare resource utilisation. [file BJO-125-1734-s002.pdf]

Table S1. Unit costs of healthcare resource utilisation

Item	Cost (2016 INR)	Cost (2016 USD)
Induction		
Foley catheterisation	117.93	6.84
Oral misoprostol (25mcg)	4.08	0.24
Oxytocin (1ml/5U)	23.91	1.39
Magnesium Sulphate (1g/2ml)	11.74	0.68
Saline (500ml)	27.17	1.58
Ringer's lactate (500ml)	43.48	2.5
Intravenous cannula	14.13	0.82
Regular drip infusion set	54.35	3.16
Hypodermic needle	4.35	0.25
Inpatient		
Indoor (per day)	21.74	1.26
Food (per day)	10.87	0.63
General ward nursing (per day)	108.69	6.31
Delivery & theatre Vaginal delivery	434.76	25.25
Operative vaginal (Forceps)	217.38	12.62
Artificial rupture of membranes	217.38	12.62
Minor procedure: perineal injury repair, repair of perineal trauma	217.38	12.62
Manual removal of placenta	217.38	12.62
Caesarean section	1086.91	63.12
Epidural analgesia	815.18	47.34
Local anaesthesia	217.38	12.62
Critical care costs		
Oxygen administration (per day)	108.69	6.31
Ventilator (per day)	217.38	12.62
Intensive care unit (per day)	217.38	12.62
Special care baby unit (per day)	217.38	12.62
Blood transfusion	489.11	28.4
Platelet transfusion	489.11	28.4
Plasma transfusion	489.11	28.4
Anti-hypertensives Aldomet (250mg)	3.59	0.21
Amlokind (2.5mg)	4.35	0.25
Aten (50mg)	2.39	0.14
Labetalol (100mg)	17.39	1.01
Nifedipine (10mg)	1.63	0.09
Antibiotics		
Amoxicillin IV (500mg)	1.30	0.08
Ciffran IV (200mg)	20.87	1.21
Ciffran 500MD	2.61	0.15
Metrodinazole IV (400mg)	15.98	0.93
Ofloxacin + Ornidazole	4.67	0.27
Taxim IV (1g)	30.87	1.79
Analgaesics		
Paracetamol (500mg)	0.82	0.05

Anti-spasmodics Drotin IV	15.54	0.9
Epidocin IV	13.59	0.79
Nutritional supplements Hovit drops	26.09	1.51
Hovit syrup	26.09	1.51
Ossopan syrup	50.00	2.9
