# Supplementary material for: Foley catheter vs. oral misoprostol to induce labour among hypertensive women in India: a cost‐consequence analysis alongside a clinical trial
Source: BJOG. 2018 Jun 22;125(13):1734–42. doi: 10.1111/1471-0528.15285 (PMC6282740; doi:10.1111/1471-0528.15285)
Supplement: Supplementary file 3 — Table S2. Comparison of healthcare costs for Foley catheterisation and oral misoprostol 25 mcg.■ [file BJO-125-1734-s003.pdf]

Table S2. Comparison of healthcare costs for Foley catheterisation and oral Misoprostol 25mcg

	Induction ($USD)		Delivery ($USD)		Hospital Stay ($USD)		Neonatal ($USD)		Total ($USD)	
	Foley	*Miso*	*Foley*	*Miso*	*Foley*	*Miso*	*Foley*	*Miso*	*Foley*	*Miso*
Gestational Age										
<32 weeks (n=5)	34.9	10.5	46.2	25.2	59.3	55.4	36.5	62.2	176.9 [95% CI 118.6-231.8]	153.4 [95% CI 114.8-194.8]
32-36 weeks (n=120)	27.8	14.3	49.5	47.7	69.1	53.2	23.1	10.1	169.3 [95% CI 141.8-199]	125.4 [95% CI 110.4-139.9]
37+ weeks (n=476)	25.6	12.9	53.5	51.3	48.4	46.2	1.2	4.3	128.7 [95% CI 125.0-132.4]	114.6 [95% CI 109.9-119.6]
Bishop's Score at Time of Induction										
≥3 (n=514)	25.5	12.7	51.7	50.1	48.7	46.1	3.3	4.9	129.2 [95% CI 124.3-133.1]	113.9 [95% CI 107.8-118]
<3 (n=84)	29.3	16.2	57.9	52.0	71.7	58.7	17.9	12.4	176.8 [95% CI 136.4-213.1]	139.2 [95% CI 124.4-152.4]
Study Recruitment Site										
GMC (n=300)	29.6	15.4	51.9	49.9	57.8	50.1	8.8	5.1	148.1 [95% CI 136.4-165.6]	120.6 [95% CI 114.4-128.7]
Daga (n=301)	22.6	10.9	53.4	50.8	46.9	45.4	2.4	6.7	125.3 [95% CI 121.2-129.9]	113.9 [95% CI 106.7-119.6]
